# Efficacy of a novel small-caliber therapeutic endoscope in peroral endoscopic myotomy for esophageal motility disorders: a propensity score matching analysis

**DOI:** 10.1007/s10388-025-01107-w

**Published:** 2025-02-04

**Authors:** Hirofumi Abe, Shinwa Tanaka, Hiroya Sakaguchi, Chise Ueda, Masato Kinoshita, Hitomi Hori, Tatsuya Nakai, Tetsuya Yoshizaki, Shinya Hoki, Hiroshi Tanabe, Satoshi Urakami, Takashi Toyonaga, Yuzo Kodama

**Affiliations:** 1 Division of Gastroenterology, Department of Internal Medicine, Kobe University Graduate School of Medicine, 7-5-1 Kusunoki-cho, Chuo-ku, Kobe, Hyogo 650-0017 Japan; 2 Department of Endoscopy, Kobe University Hospital, Kobe, Japan

**Keywords:** Esophageal motility disorders, Achalasia, Myotomy, Propensity score, Endoscopy

## Abstract

**Background:**

EG-840TP is a novel small-caliber therapeutic endoscope with a large working channel. We aimed to evaluate the treatment outcomes of peroral endoscopic myotomy using EG-840TP compared to those using a conventional therapeutic endoscope (GIF-H290T).

**Methods:**

Patients who underwent peroral endoscopic myotomy for achalasia and non-achalasia esophageal motility disorders were enrolled between March 2021 and March 2023. Procedure times and other treatment outcomes were compared between patients treated with EG-840TP and GIF-H290T using propensity score matching analysis. In the subgroup analysis, patients were divided into subsets based on myotomy length, morphology, esophageal dilation, and operator skill, and the procedure time was compared between the matched groups.

**Results:**

A total of 154 patients were enrolled in this study, and 39 patients treated using each type of scope were matched. The EG-840TP group tended to have a shorter procedure time than the GIF-H290T group. There were no significant differences between the groups in terms of short-term clinical success or perioperative adverse events. In the subgroup analysis, the procedure time of the EG-840TP group was significantly shorter than that of the GIF-H290T group when patients had a straight esophagus (44 min vs. 54 min, p = 0.0015) and the operator was a non-expert (49 min vs. 64 min, p = 0.031).

**Conclusions:**

POEM using EG-840TP showed procedure time, clinical success, and adverse events equivalent to those of a conventional therapeutic endoscope. However, EG-840TP potentially contributed to a shorter procedure time in patients with a straight esophagus or in non-expert operators than GIF-H290T.

## Introduction

Peroral endoscopic myotomy (POEM) was developed by Inoue et al. [1] in 2010 for the treatment of achalasia and other non-achalasia esophageal motility disorders (EMDs) and has become the primary treatment option for patients with minimal invasiveness and high effectiveness [2]. However, information regarding the type of endoscope that should be used during POEM for safe, effective, and efficient procedures remains limited.

To date, the safety and effectiveness of POEM using a nasal endoscope with a diameter of 5.7 mm have been reported, and, regarding the procedural aspect, it achieves a promising procedure time because it requires a narrow submucosal tunnel and a small mucosal incision [3]. However, it has not been widely used because its low stiffness leads to poor manipulation, and the availability of devices, including endoknife and coagulation forceps, is limited owing to its small working channel (2.2 mm diameter).

Although a conventional endoscope has an outer diameter of approximately 10 mm, a novel therapeutic endoscope, the EG-840TP produced by Fujifilm, has an outer diameter of only 7.9 mm but a working channel diameter as large as 3.2 mm [4]. Thus, EG-840TP will potentially facilitate the creation of a narrow submucosal tunnel and a small mucosal incision without restriction of the available devices and will improve work efficacy during POEM because it is stiffer than a nasal endoscope.

Therefore, we hypothesized that POEM using EG-840TP would result in a promising procedural time with effectiveness and safety equivalent to those of POEM using a conventional therapeutic endoscope. Accordingly, we aimed to evaluate the treatment outcomes, especially focusing on the procedure time, of POEM using EG-840TP compared with those of POEM using a conventional endoscope using propensity score (PS) matching [5], which can control the selection bias derived from retrospective data.

## Methods

### Patients

This study enrolled consecutive patients who underwent POEM for achalasia and non-achalasia motility disorders at our institution between March 2021 and March 2023. Patients who underwent POEM and those without a manometric diagnosis were excluded from the study. This observational study was conducted in accordance with the ethical standards of the 1975 Declaration of Helsinki. Institutional review board approval was obtained for this study (Approval No: B230242). Informed consent was obtained from the institution’s website using an opt-out system.

### Data collection and variables

The patients’ baseline characteristics, including age, sex, symptom duration, prior treatments, use of antithrombotic drugs, pretreatment Eckardt score [6], morphology [7], and dilation grade [7], were retrospectively collected from a prospectively maintained institutional database. The integrated relaxation pressure (IRP) was calculated using high-resolution manometry (HRM) (Starlet System^®^, Star Medical, Tokyo, Japan). Manometric diagnosis was based on the Chicago classification version 3.0 [8].

Intraoperative data, including the type of therapeutic endoscope used during POEM, operator skills, procedure time, approach direction, myotomy length, and technical difficulty, were also collected. The skills of the operator were classified into three categories according to their experience as an operator: beginner whose experience was < 25 cases, competent with experience ranging from 25 to 99 cases, and expert whose experience was ≥ 100 cases [9, 10]. Technical difficulty was defined as any of the following: (1) procedure time ≥ 90 min, (2) mucosal perforation, (3) pneumothorax, or (4) major bleeding [11]. Procedure time was defined as the time from the start of mucosal entry to the completion of entry closure and was subdivided into the four phases mentioned in the procedure subsection.

Postoperative data were collected at the 3-month follow-up after POEM, including the postoperative Eckardt score, presence of clinical success, and presence of clinical reflux. Clinical success was defined as a postoperative Eckardt score of ≤ 3. Clinical reflux was defined as erosive esophagitis of Los Angeles classification B or higher.

### Therapeutic endoscopes and devices

**Fig. 1:**
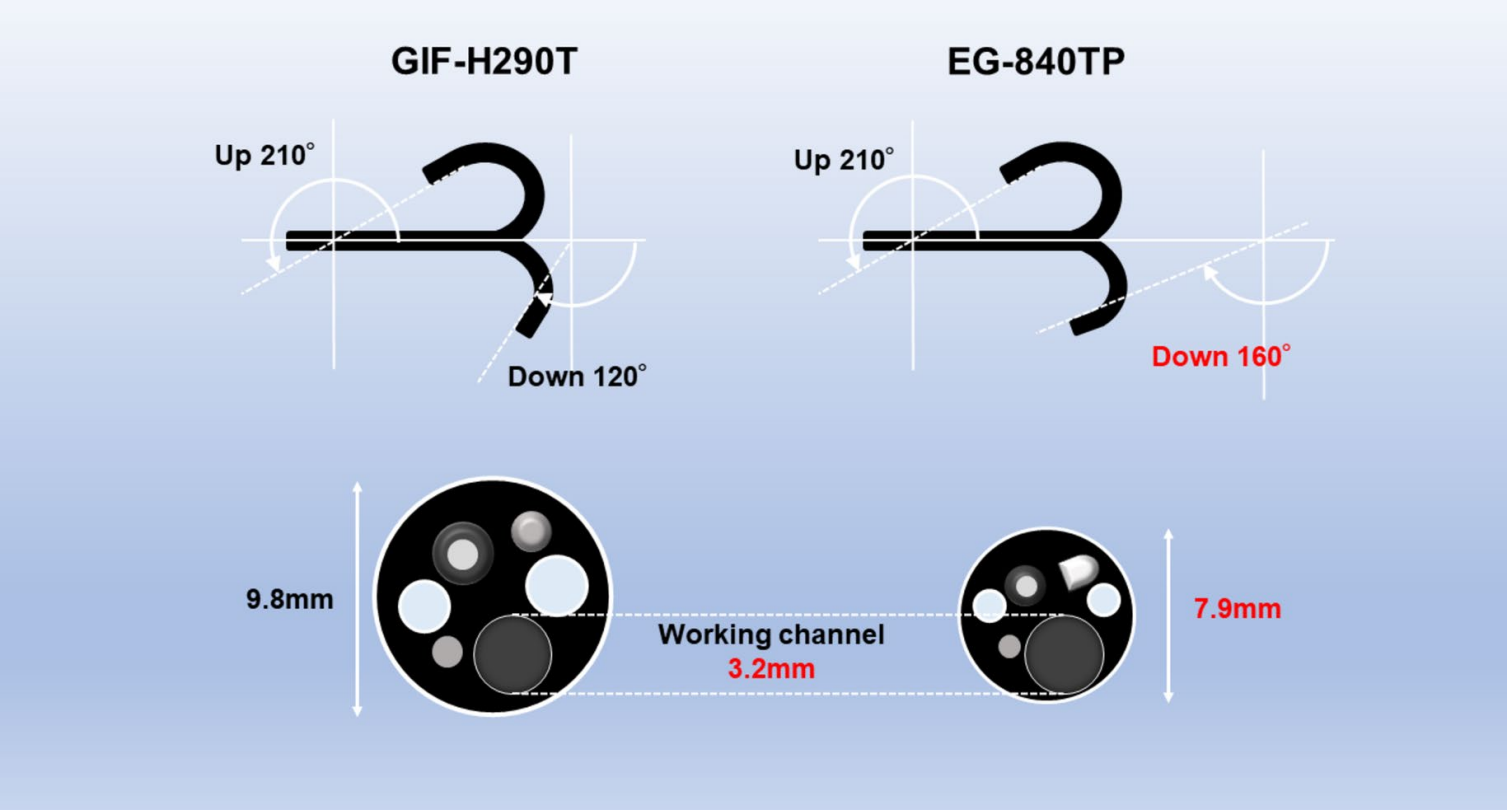
Specifications of the scopes. EG-840TP: a small-caliber therapeutic endoscope. GIF-H290T: a conventional therapeutic endoscope

POEM was performed using one of two therapeutic endoscopes: a small-caliber therapeutic endoscope (EG-840TP, Fujifilm, Tokyo, Japan) and a conventional therapeutic endoscope (GIF-H290T, Olympus, Tokyo, Japan). EG-840TP has a head diameter of 7.9 mm, a working channel diameter of 3.2 mm, a water-jet function, and working angles of 210° for up, 160° for down, 100° for right, and 100° for left. GIF-H290T has a head diameter of 9.8 mm, a working channel diameter of 3.2 mm, a water-jet function, and working angles of 210° for up, 120° for down, 100° for right, and 100° for left (Fig. 1). All procedures were performed using FlushKnife BTS 3.0 (DK2620JI-B30-, Fujifilm, Tokyo, Japan), and Coagrasper (FD-411QR; Olympus, Tokyo, Japan) was used for hemostasis, if necessary.

### Procedures

The POEM procedure was performed in four steps under general anesthesia and carbon dioxide insufflation as follows: (1) mucosal entry, (2) submucosal tunneling, (3) myotomy, and (4) closure of the entry. In most cases, myotomy was performed using the posterior approach, and the penetrating gastric vessels were identified to ensure the creation of a tunnel into the cardia.

### Postoperative management and follow-up schedule

Blood tests, chest radiography, second-look endoscopy, and barium esophagography were performed on postoperative day (POD) 1. Once the absence of adverse events (AEs) was confirmed by second-look endoscopy, clear liquid intake commenced on POD 1. Soft, solid meals were reintroduced on POD 2, followed by a normal diet on POD 4. Patients without AEs were discharged on POD 4 or 5. Medical interviews regarding achalasia-related symptoms, endoscopy, and HRM were performed at the visit 3 months after POEM.

### Propensity score matching

The variables that could affect the assignment of the therapeutic endoscope and/or outcome (i.e., procedure time) were used to calculate the PS based on the logistic regression model. Manometric diagnosis[11–13], morphology [12–14] and dilation grade [11, 15] influence the difficulty of treatment, while operator skills [9, 10] and myotomy length [14, 16, 17] are directly related to the procedure time. Furthermore, the 840TP scope facilitate submucosal insersion [4] while it has inferior stiffness to conventional scope for maneuvering in the sigmoid esophagus, which suggests that morphology and operator skills may significantly impact the choice of scope. As a result, variables including morphology, dilation grade, manometric diagnosis, operator skills, and myotomy length were selected to calculate PS. One-to-one matching with a caliper width of 0.2 was performed using nearest-neighbor matching without replacement.

### Statistical analyses

In the univariate analysis, categorical variables were expressed as counts (percentages) and analyzed using Fisher’s exact test. Continuous variables were expressed as medians (interquartile ranges) and analyzed using the *t*-test (normally distributed data) or Wilcoxon rank-sum test (skewed data). Within the matched pairs, we used the Wilcoxon signed-rank test for continuous variables and McNemar’s test for categorical variables. All calculations were performed using the R statistical software version 4.3.1 (R Foundation for Statistical Computing, Vienna, Austria) and its packages (“MatchIt”).

## Results

**Fig. 2:**
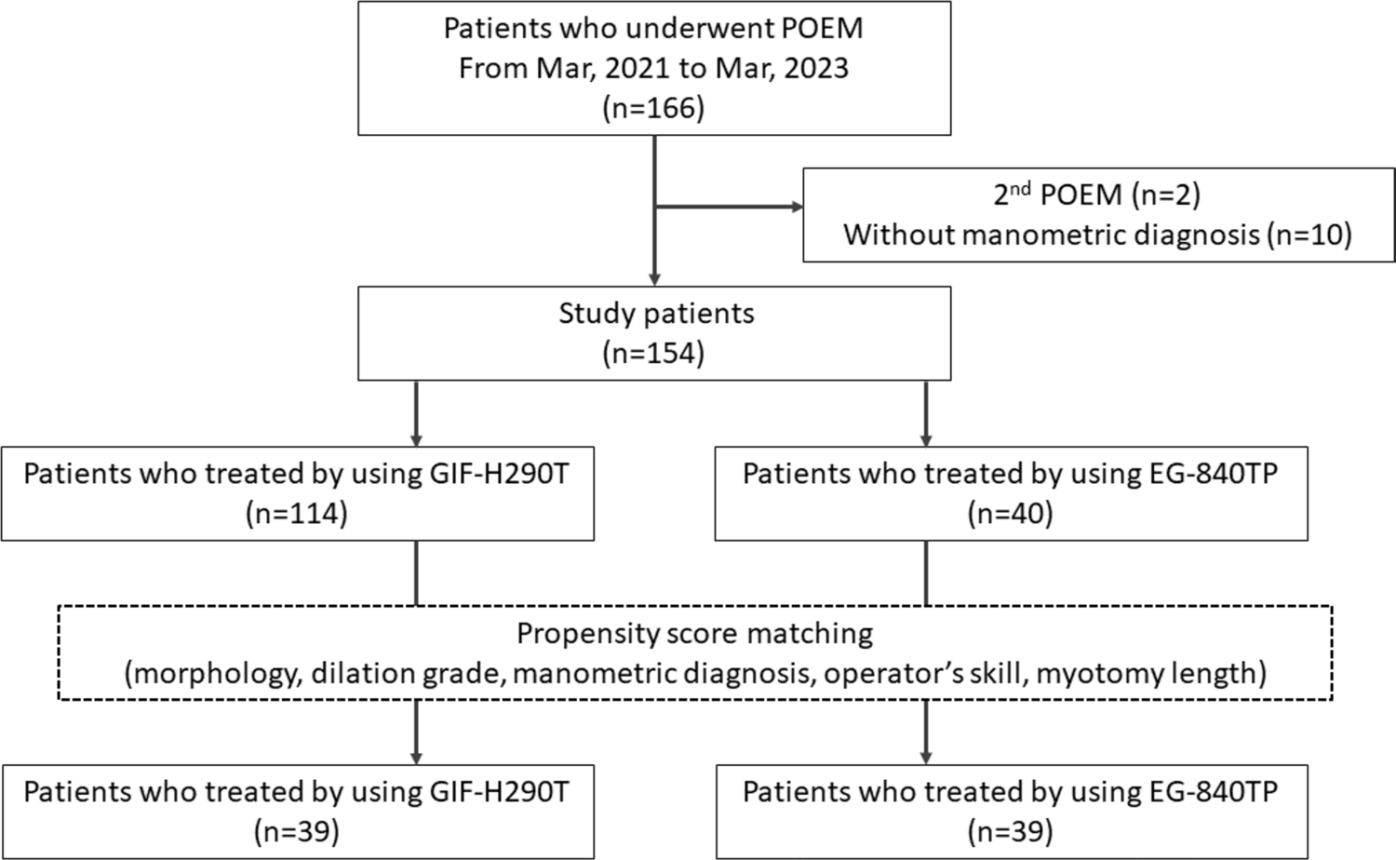
Flow diagram of patient selection for propensity score matching. POEM, peroral endoscopic myotomy

A total of 166 consecutive patients who underwent POEM for achalasia and non-achalasia EMDs between March 2021 and March 2023 were enrolled. Two patients who underwent POEM and 10 without a manometric diagnosis were excluded from this study. A total of 154 patients were eligible for this study (Fig. 2). Of the 154 study participants, 40 (26%) were treated with EG-840TP and 114 (74%) were treated with GIF-H290T. The baseline characteristics of the study participants are presented in Table 1.

**Table 1:** Baseline characteristics of 154 patients who underwent peroral endoscopic myotomy (POEM)

Age, median (IQR), years	53.5 (38–70)
Sex (male), n (%)	77 (50.0)
Duration of symptom, median (IQR), years	3.5 (1.4–8.9)
Prior treatment, n (%)	21 (13.6)
Pneumatic dilation, n (%)	21 (13.6)
Heller myotomy, n (%)	0 (0)
Antithrombotic drugs, n (%)	0 (0)
Pretreatment Eckardt score, mean (SD), points	5.90 (1.83)
Dysphagia	2.80 (0.58)
Regurgitation	1.42 (0.91)
Weight loss	1.03 (1.11)
Chest pain	0.65 (0.65)
IRP, median, median (IQR), mmHg	32.9 (24.7–42.4)
Chicago classification, n (%)	
Non-spastic disorders	140 (90.9)
Achalasia type I	107 (69.5)
Achalasia type II	27 (17.5)
EGJ outflow obstruction	6 (3.9)
Spastic disorders	14 (9.1)
Achalasia type III	10 (6.5)
Distal esophageal spasm	4 (2.6)
Jackhammer esophagus	0 (0)
Morphology, n (%)	
Straight type	121 (78.6)
Sigmoid type	20 (13.0)
Advanced sigmoid type	13 (8.4)
Dilation grade, n (%)	
Grade 1	52 (33.8)
Grade 2	95 (61.7)
Grade 3	7 (4.5)

IQR, interquartile range; SD, standard diversion; IRP, integrated relaxation pressure

Of the 40 patients who underwent POEM using EG-840TP, 39 (97.5%) were matched with patients of the same number who underwent POEM using GIF-H290T. The discriminative ability of the PS model had a C-statistic value of 0.71. The variable balance in the matched cohorts improved after PS matching (Table 2).

**Table 2:** The comparison of clinical characteristics between the EG-840TP and GIF-H290T groups in the full and propensity score-matched cohorts

	Full cohort			Matched cohort		
	EG-840TP group	GIF-H290T group	p value	EG-840TP group	GIF-H290T group	p value
	n = 40	n = 114		n = 39	n = 39	
Age, median (IQR), years	50 (33.5–58)	56 (39–71.3)	0.060	50 (32–58)	53 (36–64)	0.34
Sex (male), n (%)	17 (42.5)	60 (52.6)	0.27	16 (41.0)	21 (53.9)	0.32
Duration of symptom, median (IQR), years	2.7 (1.2–6.6)	4.0 (2.0–10.3)	0.13	2.2 (1.2–6.7)	3.4 (1.0–7.4)	0.68
Prior treatment, n (%)	7 (17.5)	14 (12.3)	0.41	6 (15.4)	5 (12.8)	0.76
Antithrombotic drugs, n (%)	2 (5.0)	8 (7.0)	0.66	1 (2.6)	2 (5.1)	0.56
Pretreatment Eckardt score, mean (SD), points	6.0 (2.0)	5.8 (1.8)	0.66	6.1 (2.0)	5.9 (1.7)	0.89
IRP, median, median (IQR), mmHg	29.4 (22.8–37.1)	34.2 (26.4–42.7)	0.15	29.3 (22.8–38.0)	37.7 (29.8–42.5)	0.76
Chicago classification, n (%)			0.30			0.56
Non-spastic disorders	38 (95.0)	102 (89.5)		37 (94.9)	38 (97.4)	
Spastic disorders	2 (5.0)	12 (10.5)		2 (5.1)	1 (2.6)	
Morphology, n (%)			0.20			0.43
Straight type	35 (87.5)	86 (75.4)		35 (89.7)	33 (84.6)	
Sigmoid type	2 (5.0)	18 (15.8)		2 (5.1)	1 (2.6)	
Advanced sigmoid type	3 (7.5)	10 (8.8)		2 (5.1)	5 (12.8)	
Dilation grade, n (%)			0.16			0.85
Grade 1	9 (22.5)	43 (37.7)		9 (23.1)	7 (18.0)	
Grade 2	28 (70.0)	67 (58.8)		28 (71.8)	30 (76.9)	
Grade 3	3 (7.5)	4 (3.5)		2 (5.1)	2 (5.1)	
Operator, n (%)			0.78			0.39
Beginner	17 (42.5)	43 (37.7)		17 (43.6)	20 (51.3)	
Competent	11 (27.5)	38 (33.3)		11 (28.2)	6 (15.4)	
Expert	12 (30.0)	33 (29.0)		11 (28.2)	13 (33.3)	
Myotomy length, median (IQR), cm	9 (7–10.8)	10.5 (9–15)	0.0016*	9 (7–11)	10 (8–12)	0.36

IQR, interquartile range; SD, standard diversion; IRP, integrated relaxation pressure

*p < 0.05

Within the matched cohort, the EG-840TP group tended to have a shorter procedure time than the GIF-H290T group (46 min vs. 55 min, p = 0.082). There were no significant differences in the prevalence of technical difficulty (2.6% vs. 2.6%, p = 1.00), short-term clinical success rate (97.4% vs. 94.4%, p = 0.61), prevalence of perioperative AEs (5.1% vs. 0%, p = 0.49), or development of clinical reflux (19.4% vs. 22.2%, p = 1.00) between the two groups (Table 3). Regarding the procedure phase, a shorter procedure time was observed in the EG-840TP group than in the GIF-H290T group, particularly at the steps of mucosal entry (2 min vs. 5 min, p < 0.0001) and closure of entry (4 min vs. 6 min, p = 0.0038). The number of clips required for closure was significantly lower in the EG-840TP group than that in the GIF-H290T group (4 vs. 5, p = 0.0034) (Table 3).

**Table 3:** The comparison of intraoperative and postoperative findings between the EG-840TP and GIF-H290T groups

	EG-840TP group	GIF-H290T group	p value
	n = 39	n = 39	
Myotomy length, median (IQR), cm	9 (7–11)	10 (8–12)	0.36
Posterior approach, n (%)	39 (100)	39 (100)	1.00
Procedure time, median (IQR) [mean (SD)], min	46 (34–61) (48.4 [17.9])	55 (41–68) (56.2 [18.6])	0.082
Making the entry site, median (IQR)	2 (2–3)	5 (3–7)	< 0.0001*
Creating a submucosal tunnel, median (IQR)	21 (13–27)	23 (17–31.8)	0.20
Myotomy, median (IQR)	17 (14–23)	19 (14–26)	0.72
Entry closure, median (IQR)	4 (2–5)	6 (4.8–8)	0.0038*
The number of clips required for closure	4 (3–5)	5 (4–6)	0.0034*
Technical difficulty, n (%)	1 (2.6)	1 (2.6)	1.00
Perioperative adverse events, n (%)	2 (5.1)	0 (0)	0.49
CRP, median (IQR), mg/dL	1.40 (0.69–2.52)	1.84 (1.16–2.72)	0.16
	n = 38	n = 37	
Clinical success—3 months, n (%)	37 (97.4)	35 (94.4)	0.61
	n = 36	n = 36	
Postoperative Eckardt score—3 months, mean (SD), points	0.72 (0.91)	1.13 (1.25)	0.10
	n = 23	n = 28	
Postoperative IRP—3 months, median (IQR), mmHg	13.6 (9.0–19.2)	14.6 (11.4–21.3)	0.19
	n = 36	n = 36	
Postoperative clinical reflux (LA grade B, C, D) –3 months, n (%)	7 (19.4)	8 (22.2)	1.00

IQR, interquartile range; SD, standard diversion; IRP, integrated relaxation pressure, *p < 0.05

In the subgroup analysis, the procedure time was significantly shorter in the EG-840TP group than in the GIF-H290T group among patients with a straight morphology (44 min vs. 54 min, p = 0.0015) and those who were treated by non-expert operators (49 min vs. 64 min, p = 0.031) (Table 4).

**Table 4:** Subgroup analysis of procedure time in terms of manometric diagnosis, operator skill, morphology, dilation grade, and myotomy length

	EG-840TP group	GIF-H290T group	p value
	n = 39	n = 39	
Procedure time, median (IQR), min			
Straight type (n = 68)	44 (33–57)	54 (42.5–67.5)	0.0015*
Sigmoid type or advanced Sigmoid type (n = 10)	72.5 (62.8–99.5)	66.5 (29.8–76.8)	0.46
Procedure time, median (IQR), min			
Dilation grade 1 (n = 16)	46 (39.5–59)	48 (45–64)	0.42
Dilation grades 2 and 3 (n = 62)	45 (32–62)	56.5 (39.3–69.5)	0.12
Procedure time, median (IQR), min			
Beginner and competent (n = 54)	49 (32.5–62.5)	64 (46–71.3)	0.031*
Expert (n = 24)	39 (37–51)	44 (34.5–57.5)	0.77
Procedure time, median (IQR), min			
Myotomy length < 10 cm (n = 39)	40 (31.8–51.8)	46 (34.5–56.5)	0.42
Myotomy length 10 cm (n = 39)	60 (37.5–69)	66 (45–76.8)	0.16

IQR, interquartile range, *p < 0.05

## Discussion

Several innovative approaches, such as an endoknife with a water-jet function [18] and short myotomy [16, 17], have been proposed to facilitate the POEM procedure resulting in shorter procedure times. However, no studies have focused on whether a specific therapeutic endoscope contributes to a shorter procedure time during POEM than a conventional therapeutic endoscope. Therefore, this is the first study to focus on the efficacy of a specific therapeutic endoscope in POEM compared with a conventional therapeutic endoscope. In this PS matching analysis, we found that EG-840TP tended to have a shorter procedure time during POEM than GIF-H290T, in addition to the equivalent efficacy and safety of GIF-H290T. The efficacy of EG-840TP in terms of procedure time was significant when the patient had a straight esophagus and the operator was a non-expert. Additionally, in the procedural phase, EG-840TP contributed to a promising procedure time, especially in terms of mucosal entry and entry closure.

Although EG-840TP tended to reduce the procedure time, there was no significant difference in the procedure time between the EG-840TP and GIF-H290T groups. We could not determine whether the lack of statistical significance was due to the absence of a difference between endoscopes or insufficient statistical power. However, based on the results of this study, we conclude that the use of EG-840TP is not recommended for all types of EMDs and operators. However, this study provides information on those who would benefit significantly from the EG-840TP. Non-expert operators benefited significantly from EG-840TP. This may indicate that EG-840TP, with a thin tip and wide downward angle, facilitates submucosal insertion and closure of the entry site, which is sometimes challenging for non-expert operators [4]. This was also consistent with the results of the present study, which showed that EG-840TP significantly contributed to reduced procedure time, especially when making a mucosal incision and closing the entry. Patients with a straight esophagus benefited significantly from EG-840TP. This may reflect the fact that the stiffness of EG-840TP is inferior to that of the conventional scope. Maintaining the axis of the scope and manipulating it is challenging in a non-straight esophagus because of its reduced stiffness, resulting in EG-840TP failing to achieve promising procedure times in such cases. This was consistent with the finding that the procedure time in the EG-840TP group was longer than that in the GIF-H290T group among patients with a sigmoid esophagus or an advanced sigmoid esophagus, although a significant difference was not observed. Given these results, if we provide recommendations regarding the use of 840TP in POEM, non-difficult cases of St-type esophagus treated by non-expert operators are considered the most appropriate candidates.

Before starting this study, we estimated that its small-caliber shaft leads to narrow tunneling, in which less tissue is dissected, based on a previous report [3], and contributes to the promising procedure time during POEM using EG-840TP. However, interestingly, EG-840TP did not significantly contribute to the promising procedure time in submucosal tunneling but did result in mucosal entry and closure of the entry. This result indicates that the advantage of a small-caliber scope in terms of procedure time is mostly derived from its capability to facilitate entry management, including submucosal insertion and entry closure. Therefore, during POEM for patients with a severely dilated esophagus whose muscularis mucosa is estimated to be thick and who have difficulty in both the creation and closure of the entry, EG-840TP is likely to contribute to a promising procedure time if the esophagus is straight and the axis of the scope can be maintained.

Regarding the efficacy and safety of POEM, no significant differences were observed between EG-840TP and GIF-H290T in terms of short-term clinical success, IRP, perioperative AEs, or postoperative clinical reflux. Some surgeons may consider passing a therapeutic scope with a larger diameter through the cardia to improve passage thorough the esophagogastric junction (EGJ). However, the results of this study indicate that the scope diameter is not important for improving passage. Therefore, it is unnecessary to be uncertain when using a small-caliber scope for fear that the small caliber affects the postoperative passage of the EGJ. However, considering the high safety and clinical success rates of POEM, further studies with larger sample sizes are required to evaluate its safety and efficacy.

Morphology, dilation grade, manometric diagnosis, operator skills, and myotomy length were selected as adjustment variables for PS calculation. These variables were considered to be associated with procedure time and technical difficulty [9–17]. Although the adjusting variables should be the preoperative variable and myotomy length was not a preoperative variables, it was selected as the adjusting variable because myotomy length strongly affects procedure time. The determination of myotomy length was mainly dependent on the preoperative determination of the entry site. After PS matching, the variable balance in the matched cohort improved. The proportion of patients after matching differed in some clinical characteristics from that before matching. The proportion of patients with spastic EMDs was lower after matching than before matching. Therefore, the applicability of the results of this study is limited to the general population.

This study had some limitations. First, this study had a retrospective design; therefore, both information and selection biases were not completely excluded, even when the PS-matching approach was used. Second, the matched cohort did not completely represent the general population. Third, the sample size could have been significantly small for the evaluation of procedure time between the cohorts. Fourth, the significant results of subgroup analyses in PS matching should be interpreted cautiously, as they are subject to biases from multiplicity in multiple testing. Although a prospective study with a sufficient sample size based on the effect size from this study is required to overcome these limitations, the results of this retrospective study provide suggestive information regarding the use of novel therapeutic endoscopy during POEM for those who engage in the clinical practice of EMDs.

In conclusion, EG-840TP significantly contributed to a shorter procedure time in patients with a straight esophagus and non-expert operators. However, the efficacy of the procedure does not significantly affect patients or operators. Therefore, EG-840TP is recommended for specific patients and surgeons.

## Data Availability

The data supporting the findings of this study can be provided upon reasonable request to the corresponding author. However, the data are not publicly available due to patient privacy concerns or ethical restrictions.
